# Venoarterial Extracorporeal Membrane Oxygenation in Cardiogenic Shock to Ventricular Assist Device or Heart Transplantation - Where Are We?

**DOI:** 10.21470/1678-9741-2023-0960

**Published:** 2023

**Authors:** Alvaro Perazzo, Lisa Anderl, Ricardo de Carvalho Lima, Dominik Wiedemann, Roberto Lorusso

**Affiliations:** 1 Department of Cardiac Surgery, Pronto-Socorro Cardiológico de Pernambuco (PROCAPE), Universidade de Pernambuco, Recife, Pernambuco, Brazil.; 2 Cardio-Thoracic Surgery Department, Heart and Vascular Center, Maastricht University Medical Center, Cardiovascular Research Institute Maastricht, Maastricht University, Maastricht, The Netherlands.; 3 Department of Cardiac Surgery, Medical University of Vienna, Vienna, Austria.

Cardiogenic shock (CS) is a complex, multifactorial, and highly morbid condition requiring interdisciplinary expertise and state-of-the-art management. Despite advances in therapeutic options, CS’ 30-day mortality remains high, around 40-50% in contemporary randomized trials. CS is caused by impaired myocardial contractility, resulting in reduced cardiac output, end-organ hypoperfusion, and hypoxia. The inability to meet the body’s metabolic demands due to diminished cardiac output leads to insufficient tissue perfusion^[1]^. Acute myocardial infarction is the most common cause of CS (7-10%)^[2,3]^. Various conditions eventually lead to CS, most commonly valvular regurgitation, ischemic and non-ischemic cardiomyopathy, pericardial disease, and arrhythmia. The American Heart Association emphasizes the importance of early monitoring and initial stabilization prior to invasive management^[1]^. A variety of mechanical circulatory support (MCS) devices have been introduced with the goal of providing hemodynamic support and improving outcomes. The basic concept is that support and decompression of the ventricle lead to reduced myocardial stress and consumption of oxygen while increasing end-organ perfusion. Venoarterial extracorporeal membrane oxygenation (VA-ECMO) offers immediate circulatory support and concomitant gas exchange for patients with left and right ventricular failure^[1,4]^. Despite the recognized advances of VA-ECMO (Figure 1), there are significant discrepancies in research concerning the hemodynamic implications of its long-term use. After restoring hemodynamic stability with adequate neurological and renal function, the following question arises: what is the next step after VA-ECMO - heart transplantation (HTx) or implantation of a ventricular assist device (VAD)?

**Fig. 1:**
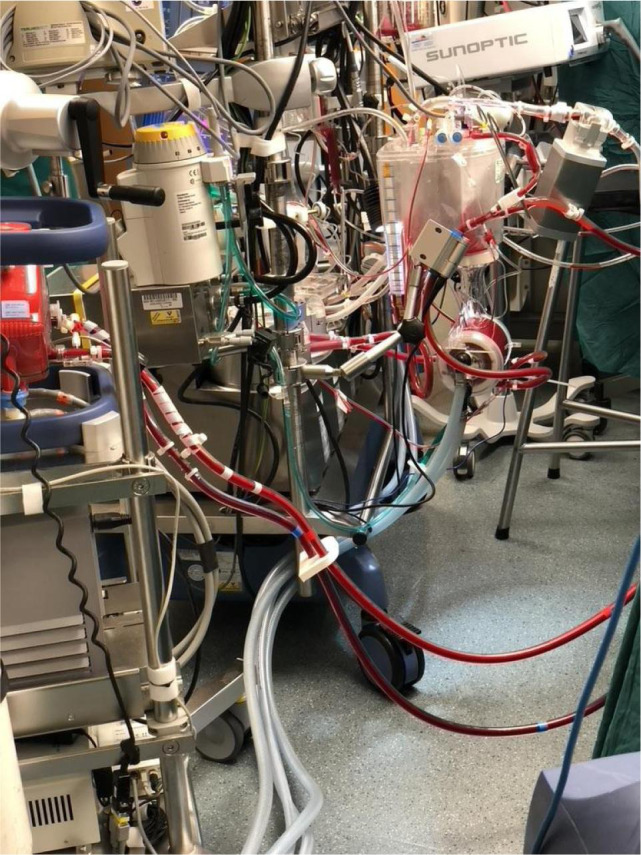
Venoarterial extracorporeal membrane oxygenation implanted.

VA-ECMO offers temporary bridge-to-recovery with restoration of normal cardiac function, bridge-to-bridge with implantation of temporary VAD, or bridge-to-destination with more durable left VAD (Figure 2) or cardiac transplantation^[5]^. Despite advances in therapeutic options for CS, the outcome and quality of life of these patients rely on multidisciplinary efforts, from technology and engineering of the device to surgical and intensive care expertise. Establishment of standardized protocols and the shock team’s multidisciplinary cooperation and shared decision-making, including evaluation of timing and strategy to escalate or de-escalate MCS, are the primary aims of care.

**Fig. 2:**
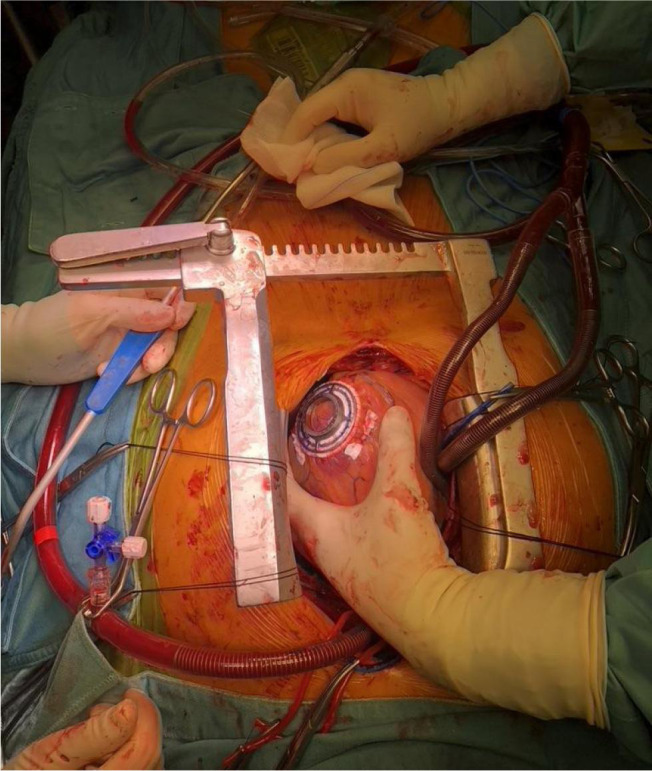
Implant of left ventricular assist device (HeartMate 3™).

Institutional protocols and algorithms are fundamental to add more surveillance and improve outcomes for these patients. In case of impossibility of left ventricular recovery with intensification of VA-ECMO, referral to the VAD team must be considered, if the metabolic conditions are resolved and neurological assessment shows no diffuse lesions^[6]^. In terms of VAD, the Impella CP® 5.0 and 5.5, intra-aortic balloon pump, TandemHeart®, HeartMate 3™, and Impella RP® provide possible options for these patients. Although VA-ECMO offers bridging to durable left VAD or HTx, the possibility of full cardiac recovery must be considered with daily cardiorespiratory tests evaluating the possibility of weaning.

If VAD therapy proves to be ineffective, it is crucial to involve the HTx team, especially when transplant requirements are met and no contraindications for HTx arise. Orthotopic HTx is an effective alternative for patients requiring long-term support. Aiming for HTx after VA-ECMO is not straightforward; it is critical that protocols and institutions are well-connected with a national or international organization managing organ donation. The scarcity of donors, primary graft dysfunction, and other complications following transplantation are limiting factors of this procedure. At the same time, donation after circulatory arrest, hypothermic preservation, and *ex-vivo* heart perfusion are developing advances in HTx surgery that may allow for organ procurement from greater distances and prevention of early transplant failure^[7,9]^. Some centers in Australia and Europe have pioneered HTx after cardiac death in response to the critical demand for donor hearts^[8]^. The optimal timing to refer the patient for HTx remains unclear. Due to the many challenges and importance of shared decision-making, a shock-team is essential. It is crucial to define standard protocols and regularly educate the multidisciplinary team.

In conclusion, recent developments in MCS technology have caused a paradigm shift in CS care with current consensus advocating for early use of VA-ECMO in refractory CS. VA-ECMO has progressed to the point where skilled practitioners initiate the device within minutes, providing complete cardiorespiratory support. After initial diagnosis, hemodynamic stabilization of the patient is essential. The decision-making to progress to more durable devices or HTx requires interdisciplinary teamwork by means of a shock team consisting of both HTx and VAD participants. There is a scarcity of current data on trends, results, and exit strategies of patients undergoing VA-ECMO for CS. Numerous options for escalating or de-escalating MCS exist, but determining the exact timing remains a challenging and crucial task. Further studies, protocols, and definitions of criteria are necessary to propose algorithms for improved patient management.

## References

[1] Henry TD, Tomey MI, Tamis-Holland JE, Thiele H, Rao SV, Menon V, Klein DG, Naka Y, Piña IL, Kapur NK, Dangas GD, American Heart Association Interventional Cardiovascular Care
Committee of the Council on Clinical Cardiology; Council on
Arteriosclerosis, Thrombosis and Vascular Biology; and Council on
Cardiovascular and Stroke Nursing (2021). Invasive Management of Acute Myocardial Infarction Complicated by
Cardiogenic Shock: A Scientific Statement From the American Heart
Association. Circulation.

[2] Harjola VP, Lassus J, Sionis A, K.ber L, Tarvasm.ki T, Spinar J, Parissis J, Banaszewski M, Silva-Cardoso J, Carubelli V (2015). CardShock Study Investigators; GREAT Network. Clinical picture
and risk prediction of short-term mortality in cardiogenic
shock. Eur J Heart Fail.

[3] Kolte D, Khera S, Aronow WS, Mujib M, Palaniswamy C, Sule S, Jain D, Gotsis W, Ahmed A, Frishman WH (2014). Trends in incidence, management, and outcomes of cardiogenic
shock complicating ST-elevation myocardial infarction in the United
States. J Am Heart Assoc.

[4] Winiszewski H, Guinot PG, Schmidt M, Besch G, Piton G, Perrotti A, Lorusso R, Kimmoun A, Capellier G. (2022). Optimizing PO2 during peripheral veno-arterial ECMO: a narrative
review. Crit Care.

[5] Lorusso R, Shekar K, MacLaren G, Schmidt M, Pellegrino V, Meyns B, Haft J, Vercaemst L, Pappalardo F, Bermudez C, Belohlavek J, Hou X, Boeken U, Castillo R, Donker DW, Abrams D, Ranucci M, Hryniewicz K, Chavez I, Chen YS, Salazar L, Whitman G (2021). ELSO Interim Guidelines for Venoarterial Extracorporeal Membrane
Oxygenation in Adult Cardiac Patients. ASAIO J.

[6] Kowalewski M, Zieliński K, Gozdek M, Raffa GM, Pilato M, Alanazi M, Gilbers M, Heuts S, Natour E, Bidar E, Schreurs R, Delnoij T, Driessen R, Sels JW, van de Poll M, Roekaerts P, Pasierski M, Meani P, Maessen J, Suwalski P, Lorusso R (2021). Veno-Arterial Extracorporeal Life Support in Heart Transplant and
Ventricle Assist Device Centres. Meta-analysis. ESC Heart Fail.

[7] DeFilippis EM, Khush KK, Farr MA, Fiedler A, Kilic A, Givertz MM (2022). Evolving Characteristics of Heart Transplantation Donors and
Recipients: JACC Focus Seminar. J Am Coll Cardiol.

[8] Rajab TK, Singh SK (2018). Donation After Cardiac Death Heart Transplantation in America Is
Clinically Necessary and Ethically Justified. Circ Heart Fail.

[9] Tong CKW, Khush KK (2021). New Approaches to Donor Selection and Preparation in Heart
Transplantation. Curr Treat Options Cardiovasc Med.

